# Delivery of Bone Marrow-Derived Mesenchymal Stem Cells Improves Tear Production in a Mouse Model of Sjögren's Syndrome

**DOI:** 10.1155/2017/3134543

**Published:** 2017-03-02

**Authors:** Hema S. Aluri, Mahta Samizadeh, Maria C. Edman, Dillon R. Hawley, Helene L. Armaos, Srikanth R. Janga, Zhen Meng, Victor G. Sendra, Pedram Hamrah, Claire L. Kublin, Sarah F. Hamm-Alvarez, Driss Zoukhri

**Affiliations:** ^1^Department of Comprehensive Care, Tufts University School of Dental Medicine, Boston, MA, USA; ^2^Department of Ophthalmology, USC Roski Eye Institute, Keck School of Medicine of University of Southern California, Los Angeles, CA, USA; ^3^University of Southern California School of Pharmacy, Los Angeles, CA, USA; ^4^Department of Ophthalmology, Tufts University School of Medicine, Boston, MA, USA; ^5^Center for Translational Ocular Immunology, Department of Ophthalmology, Tufts University School of Medicine, Boston, MA, USA

## Abstract

The purpose of the present study was to test the potential of mouse bone marrow-derived mesenchymal stem cells (BD-MSCs) in improving tear production in a mouse model of Sjögren's syndrome dry eye and to investigate the underlying mechanisms involved. NOD mice (*n* = 20) were randomized to receive i.p. injection of sterile phosphate buffered saline (PBS, control) or murine BD-MSCs (1 × 10^6^ cells). Tears production was measured at baseline and once a week after treatment using phenol red impregnated threads. Cathepsin S activity in the tears was measured at the end of treatment. After 4 weeks, animals were sacrificed and the lacrimal glands were excised and processed for histopathology, immunohistochemistry, and RNA analysis. Following BD-MSC injection, tears production increased over time when compared to both baseline and PBS injected mice. Although the number of lymphocytic foci in the lacrimal glands of treated animals did not change, the size of the foci decreased by 40.5% when compared to control animals. The mRNA level of the water channel aquaporin 5 was significantly increased following delivery of BD-MSCs. We conclude that treatment with BD-MSCs increases tear production in the NOD mouse model of Sjögren's syndrome. This is likely due to decreased inflammation and increased expression of aquaporin 5.

## 1. Introduction

Sjögren's syndrome is an autoimmune disease which chronically affects the lacrimal and salivary glands, leading to dry eye disease and dry mouth (xerostomia) [1, 2]. Dry eye disease caused by Sjögren's syndrome is associated with corneal injury and recurrent eye infections that may lead to impairment of vision. Furthermore, symptoms of dry eye result in loss of quality of life comparable to that caused by angina. Although the etiology of Sjögren's syndrome is not yet clear, B lymphocyte hyperreactivity, autoantibody production, and T cell lymphocytic infiltration to exocrine glands and other organs have been observed in patients diagnosed with Sjögren's syndrome [3, 4].

In recent years, there has been an emerging interest in stem cells therapy for autoimmune diseases. One group of stem cells that were originally isolated from human bone marrow, but are found in many other adult tissues, are mesenchymal stem cells (MSCs), also termed multipotent stromal cells. MSCs are nonhematopoietic, multipotent progenitor cells that can differentiate in vitro and in vivo to mesenchymal lineages. In addition, MSCs have immunoregulatory capabilities that affect both adaptive and innate immunity. MSCs have been reported to secrete interleukin 6 (IL-6) and macrophage-colony stimulating factor to suppress T and B lymphocyte proliferation [5–7] along with the release of anti-inflammatory and antiapoptotic molecules that protect damaged tissues [8]. Addition of MSCs to in vitro activated T cell cultures resulted in decreased production of proinflammatory cytokines and increased levels of anti-inflammatory cytokines [6, 9–11]. Even though in vitro and in vivo studies support the therapeutic efficacy of MSCs, the mechanisms by which MSCs exert immunomodulatory and tissue repair processes are still not completely understood.

Xu et al. showed that treatment with BD-MSCs, or umbilical cord derived MSCs, suppressed autoimmunity and restored salivary gland secretory function both in a mouse model and in Sjögren's syndrome patients [12]. In another study, Khalili et al. found that CD45^−^/TER119^−^ BD-MSCs injected into female NOD mice restored normal salivary secretion [13]. From several studies involving MSC therapy in autoimmune diseases, it is apparent that MSCs are not replacing the tissue by differentiating into specific tissue lineage but they are exerting a complex pattern of immunomodulatory, anti-inflammatory, and regenerative signaling effects that help in attenuating the disease condition [6]. At the time of writing this article, there are 311 registered clinical trials in progress using MSCs (http://www.clinicaltrials.gov/), including one study for Sjögren's syndrome.

Our lab has performed extensive research on the mechanisms involved in dry eye and dry mouth in human and animal models of Sjögren's syndrome [14–19]. We reported that stem/progenitor cells were present in the murine lacrimal gland and the number of these cells increased during the repair phase after experimentally induced inflammation [16]. We also have found evidence that MSCs in murine lacrimal glands can be activated during tissue repair [20]. Since BD-MSCs display definite immunomodulation and immunosuppressive properties as well as regenerative potential, there is a strong rationale for using MSC-based therapy for treatment of Sjögren's syndrome dry eye. Thus, the purpose of the present study was to test the hypothesis that a single injection of BD-MSCs will restore normal tear production in a mouse model of Sjögren's syndrome.

## 2. Materials and Methods

### 2.1. Materials

Iscove's Modified Dulbecco's Medium (IMDM), gentamicin, penicillin, streptomycin, heat-inactivated fetal bovine serum (FBS), L-glutamine, trypsin-EDTA, and trypsin replacement (TrypLE Express) were obtained from Invitrogen (Carlsbad, CA). Costar culture treated plates were from Corning Life Sciences (Lowell, MA). The miRNeasy isolation kit and OneStep RT-PCR Kit were purchased from Qiagen (Valencia, CA). Primers were designed using NCBI/Primer-BLAST (http://www.ncbi.nlm.nih.gov/tools/primer-blast/). Phenol red impregnated cotton threads were obtained from Zone-Quick, Lacrimedics (Eastsound, WA). Paraplast™ plus tissue embedding medium, eosin, and modified hematoxylin were obtained from Fisher Healthcare (Houston, TX). For the immunohistochemistry experiments, the antigen retrieval solution and DAPI were from Vector Laboratories (Burlingame, CA) whereas the donkey serum was purchased from Jackson ImmunoResearch (Westgrove, PA). Bovine serum albumin (BSA, fraction V) was purchased from Fisher Scientific (Waltham, MA). The following primary antibodies were used: purified rat anti-mouse Foxp3 (1 : 100; clone FJK-16s, eBioscience, San Diego, CA) and rabbit polyclonal to Foxp3 (1 : 100, Abcam, Cambridge, MA). Secondary antibodies were as follows: Alexa Fluor® 488 donkey anti-rat IgG and Alexa Fluor® 488 donkey anti-rabbit IgG (1 : 100; Invitrogen, Eugene, OR). For CTSS assay, the Cathepsin S Activity Fluorometric Assay Kit (BioVision, Milpitas, CA) was used.

### 2.2. Animals and Treatments

The studies described herein were performed at two sites, Tufts University and the University of Southern California. Male NOR/LtJ (referred to as NOD) mice were purchased or bred in house from breeding pairs purchased from the Jackson Laboratories (Bar Harbor, ME). All experiments were performed in accordance with the Association of Research in Vision and Ophthalmology (ARVO) Statement for the Use of Animals in Ophthalmic and Vision Research and were approved by the Tufts Medical Center and University of Southern California Animal Care and Use Committees.

BD-MSCs isolated from C57BL/6-Tg (UBC-GFP) mice (a generous gift from Texas A&M Health Science Center College of Medicine Institute for Regenerative Medicine at Scott & White) were injected intraperitoneally into NOD mice. The protocols for isolation and propagation of mouse BD-MSCs were published by Peister et al. [21]. The published culture protocol was used for propagation of the donated BD-MSCs. Thirteen-week-old male NOD mice (*n* = 10 per group) were randomized to receive PBS (control) or BD-MSCs (1 × 10^6^ cells/mouse in 0.2 mL PBS) intraperitoneally. Following 4 weeks of treatment, the animals were sacrificed and the exorbital lacrimal glands were removed and divided into two equal pieces. One piece of the lacrimal gland was fixed, embedded in paraffin, and used for histopathology and immunohistochemistry studies. From the second piece of the lacrimal gland, total RNA was extracted and used for PCR analyses.

### 2.3. Tear Measurement and Cathepsin S Assay

Mice were lightly anesthetized using isoflurane and tear production was measured using phenol red impregnated cotton threads, as previously described [22]. The threads were held with jeweler forceps and applied in both eyes at the lateral canthus of the ocular surface for 10 sec. Upon contact with the tears, the color of the thread changes from yellow to red. The length of the thread that changed color was then measured, in millimeters, using a dissecting microscope. Following 4 weeks of treatment, stimulated tears were collected and used for cathepsin S (CTSS) assay. Briefly, mice were anesthetized with ketamine and xylazine and then the eye was washed with a drop of eye irrigating solution (Eyestream, Alcon). A small incision was made between the eye and ear to expose the exorbital lacrimal gland followed by the application of 3 *μ*L of a 50 *μ*M carbamylcholine solution (an analog of the parasympathetic agonist acetylcholine) topically to the gland, 3 times in 5-minute intervals. Tear fluid was then collected with a 2 *μ*L glass capillary at the lateral canthus of the eye for a total of 15 minutes. Tear fluid was analyzed for CTSS activity using a commercially available kit (BioVision) according to the manufacturer's instructions. Briefly, 100 *μ*L of diluted tear sample with CS reaction buffer and 2 *μ*L of the CTSS substrate Z-VVR-AFC (the tripeptide Val-Val-Arg conjugated to 7-amino-4-trifluoromethyl coumarin [AFC]) were loaded into a 96-well clear plate in the presence or absence of the supplied CTSS inhibitor, followed by incubation at 37°C for 1 hour. The quantity of free AFC obtained by the reaction of CTSS with substrate was measured using a SpectraMax Gemini EM spectrofluorometer (Molecular Devices) with excitation at 400 nm and emission at 505 nm. CTSS-specific activity was calculated by subtracting CTSS activity values obtained in the presence of the inhibitor, from the values obtained in the absence of the inhibitor.

### 2.4. Histopathology and Measurement of Lymphocytic Foci Number/Size

Lacrimal glands were fixed in 4% formaldehyde in phosphate buffered saline (PBS, containing NaCl 145 mM, Na_2_HPO_4_ 7.3 mM, and NaH_2_PO_4_ 2.7 mM at pH 7.2) and embedded in paraffin. Paraffin sections of the lacrimal gland (6 *μ*m) were deparaffinized followed by rehydration in graded alcohol solutions prior to staining. For histopathology experiments, slides were processed for hematoxylin and eosin staining and lymphocytic foci were counted. Images were obtained using a Nikon UFXII microscope coupled to a SPOT™ digital camera. A lymphocytic focus was defined as a focus containing 50 or more mononuclear cells. The size and number of lymphocytic foci, in addition to the total section area, were determined in 5–8 nonconsecutive sections per gland. The percentage of the area occupied by a focus was calculated as

(1) ∑area of individual focus in a given section×100÷total section area.

The slides were masked and two individuals performed the histologic analysis as a blindfolded experiment.

### 2.5. Immunofluorescence Staining

Lacrimal glands from tissue embedded paraffin were used to prepare 6 *μ*m sections. After heat-induced antigen retrieval, the sections were permeabilized with 0.1% Triton X-100 (in PBS) for 10 min. Next, F_c_ antigen-binding fragments were blocked using 10% (w/v) normal donkey serum diluted in 1% (w/v) bovine serum albumin (BSA) in PBS, for 1 h at room temperature. Sections were then incubated with purified rat anti-forkhead box P3 (Foxp3) or rabbit anti-Foxp3 primary antibodies overnight at 4°C or for 1 hour at 37°C, respectively. Subsequently, sections were incubated with the appropriate secondary antibody, AlexaFluor® 488 donkey anti-rat IgG for 1 hour at room temperature or AlexaFluor 488 donkey anti-rabbit IgG for 1 hour at 37°C. For negative controls, incubation without the primary antibody was performed. Finally, sections were mounted in Vectashield mounting medium containing DAPI or ProLong Gold Antifade Mounting Media. Sections were examined with a fluorescence microscope to identify and count the number of Foxp3^+^ cells/focus.

### 2.6. RNA Extraction and RT-PCR/qPCR

Total RNA was extracted using the miRNeasy isolation kit (Qiagen, Valencia, CA) according to the manufacturer's protocol. Briefly, lacrimal glands were homogenized in QIAzol lysis reagent and incubated at room temperature for 5 min. To the homogenate, chloroform was added and centrifuged at 12,000 ×g at 4°C. Next, the upper aqueous layer was collected and mixed with 1.5 volumes of 100% ethanol which was run through an RNeasy MinElute spin column at 8000 ×g for 15 seconds. Buffers RWT, RPE, and 80% ethanol were run through the column, in sequence, using centrifugation at 8000 ×g for 15 seconds each time. Lastly, 20 *μ*L of RNase-free water was added to the spin column and the RNA was collected. RNA purity and quantity was analyzed using a NanoDrop 1000. The samples were then stored at −20°C until being used for downstream applications.

Purified total RNA (20 ng) was used for reverse transcription and PCR amplification with OneStep RT-PCR Kit using primers specific to C-X-C chemokine receptor type 4 (CXCR4), C-X-C motif chemokine 12 (CXCL12), tumor necrosis factor-inducible gene 6 (TSG-6), Foxp3, or glyceraldehyde 3-phosphate dehydrogenase (GAPDH) in an Applied Biosystems 2720 thermal cycler (Foster City, CA). The primers (shown in Table 1) were designed using NCBI/Primer-BLAST. The reverse transcription reaction was conducted at 52°C for 30 minutes followed by PCR according to the manufacturer's instructions. The cycling conditions were 15 minutes hot start at 95°C, followed by 25 to 30 cycles of denaturation for 40 seconds at 94°C, annealing for 40 seconds at 53°C, extension for 1 minute at 72°C, and a final extension at 72°C for 10 minutes. After amplification, the products were separated by electrophoresis on a 1.5% agarose gel, stained with ethidium bromide, and visualized under UV light.

For qPCR, total RNA was converted to cDNA using a reverse transcription kit (Thermo Scientific, Grand Island, NY) following manufacturer's protocol. qPCR was then performed using specific TaqMan primers and probes against CTSS, major histocompatibility complex II (MHC-II), matrix metalloproteinase 9 (MMP9), Rab3D, Rab27A, Rab27B, interleukin 12a (IL-12a), interferon gamma (IFN-*γ*), tumor necrosis factor alpha (TNF-*α*), aquaporin 5 (Aq5), M3R, and GAPDH as a housekeeping gene (Table 2). An ABI Open Array Real-Time PCR System (Life Technologies, Grand Island, NY) was used to read the plate and the data obtained was analyzed using the ΔΔCT method.

### 2.7. Statistical Analysis

All data are expressed as means ± standard error of the mean (SEM) and data consisting of two groups was analyzed by two-tailed Student's *t*-test using GraphPad Prism Version 5.0 software (GraphPad Software, Inc., La Jolla, CA). Values of *p* < 0.05 were considered statistically significant.

## 3. Results

### 3.1. Aqueous Tear Production Increased in BD-MSC Treated NOD Mice

**Figure 1:**
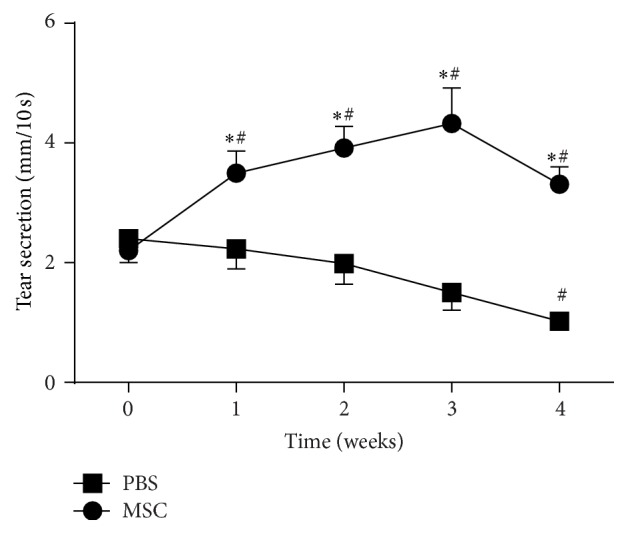
Aqueous tear production in BD-MSC treated and nontreated NOD mice. At baseline (week 0) and every week posttreatment (for 4 weeks), tear production in BD-MSC treated and PBS (control) treated mice was measured for 10 seconds using phenol red impregnated cotton threads. For statistical analyses each eye was considered separately and the data are presented as means ± SEM (*n* = 20 for each group). *∗* denotes statistically significant difference compared to control; # denotes statistically significant difference compared to baseline.

Analysis of tear production (Figure 1) every week posttreatment showed a significant increase in tear volume from the BD-MSC treated group compared to PBS treated group (control). A mean difference between MSC injected and PBS injected of 0.94, 1.58, 2.44, and 1.88 mm/10 s was observed at 1, 2, 3, and 4 weeks after injection, respectively. At each of these measurements, tear volume in MSC injected groups was also significantly increased compared to baseline, with mean differences of 1.17, 1.56, 1.94, and 0.90 mm/10 s, respectively (Figure 1). In contrast, mice treated with PBS showed a downward trend in tear volume over the total course of measurements (Figure 1) which became significant 4 weeks after injection with a mean difference in tear production of −0.98 mm/10 s.

These data suggest that a single injection of BD-MSCs leads to a sustained improvement in aqueous tears production in NOD mice, a Sjögren's syndrome animal model of lacrimal gland deficiency.

### 3.2. BD-MSC Treatment Did Not Alter CTSS Activity in Tears of NOD Mice

**Figure 2:**
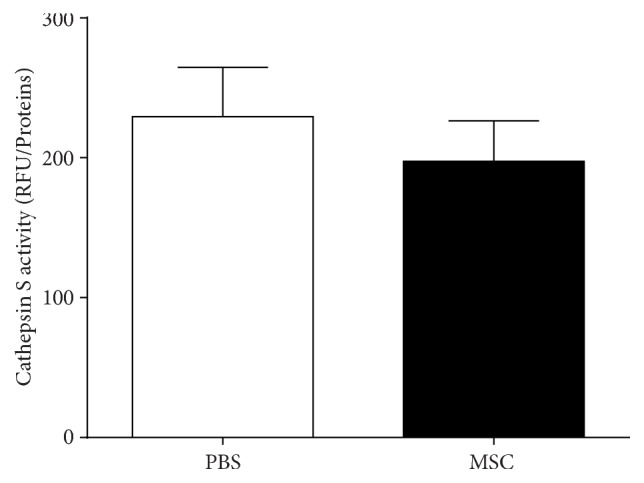
Cathepsin S activity in the tears of BD-MSC treated and nontreated NOD mice. Following 4 weeks of treatment, tears were collected from BD-MSC and PBS (control) treated mice and used to measure cathepsin S activity as described in the Methods. Data are presented as means ± SEM (*n* = 7 for each group).

The activity of the lysosomal enzyme CTSS is elevated in tears and lacrimal glands of male NOD mice with established dacryoadenitis compared to healthy BALB/c mice [23]. CTSS activity was also found to be increased in tears of Sjögren's syndrome patients when compared to patients with other autoimmune diseases, nonautoimmune dry eye, and healthy controls [24]. We measured CTSS activity in tears collected from MSC treated animals and those from PBS treated ones. As shown in Figure 2, there was a modest (13.5%), but not statistically significant, decrease in CTSS activity in tears from NOD mice treated with MSCs compared to the PBS treated group.

These data suggest that treatment with BD-MSCs did not significantly affect the activity of CTSS in lacrimal gland tear fluid of NOD mice.

### 3.3. Lymphocytic Foci Size Was Reduced in BD-MSC Injected Group

**Figure 3:**
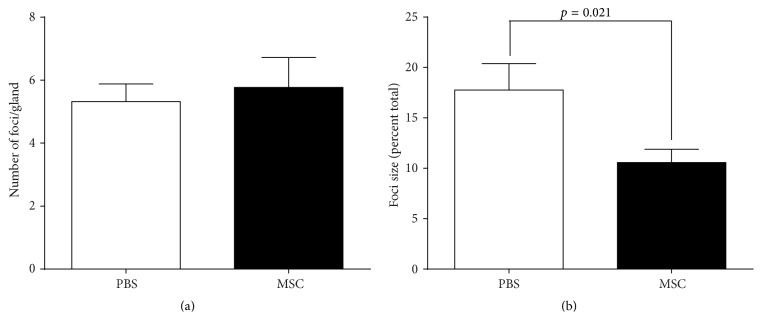
Lymphocytic foci number and size in BD-MSC treated and nontreated NOD mice. Following 4 weeks of treatment lacrimal glands were extracted and processed for paraffin embedding. The glands were sectioned and stained with hematoxylin and eosin to measure the number (a) and size (b) of lymphocytic foci. For statistical analyses each lacrimal gland was considered separately and the data are presented as means ± SEM (*n* = 20 for each group).

To examine whether BD-MSC treatment might ameliorate lymphocyte accumulation in the lacrimal glands of NOD mice, sections were stained with hematoxylin and eosin and evaluated quantitatively to measure the number and size of the lymphocytic foci (as described in Materials and Methods). As shown in Figure 3(a), there was no difference in the average number of lymphocytic foci in sections from BD-MSC treated (5.77 ± 0.95 foci/section) compared to control PBS treated (5.32 ± 0.56 foci/section) mice. In contrast, there was a statistically significant 40.5% decrease in the percentage of the area infiltrated by lymphocytes in the lacrimal glands of animals treated with BD-MSCs (10.57 ± 1.32% infiltrated) when compared to the PBS treated group (17.76 ± 2.63% infiltrated) (Figure 3(b)).

These data indicate that the size of lymphocytic foci decreased in the lacrimal glands of NOD mice treated with BD-MSCs.

### 3.4. The Number of Foxp3^+^ Lymphocytes Was Unchanged in BD-MSC Treated Animals

**Figure 4:**
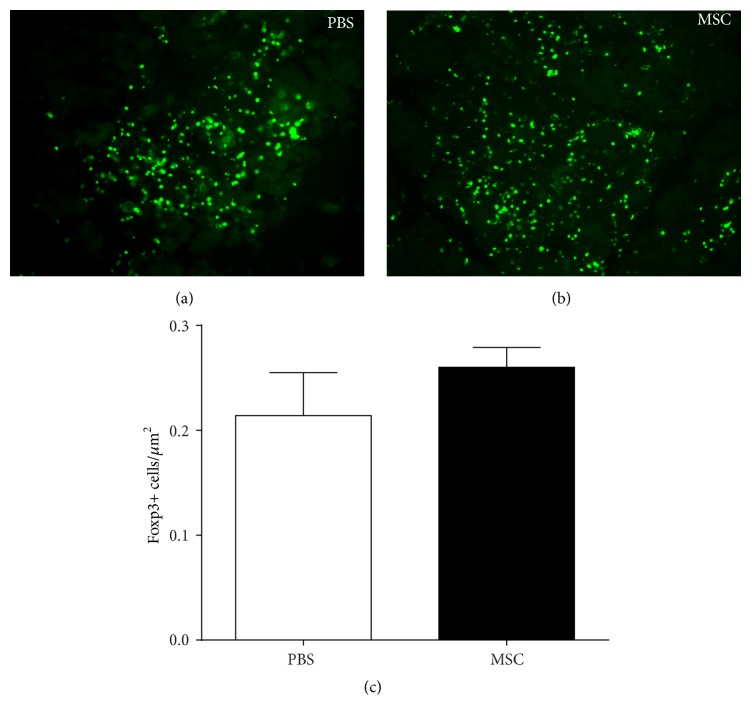
Number of Foxp3+ cells in the lymphocytic foci of BD-MSC treated and nontreated NOD mice. Following 4 weeks of treatment lacrimal glands were removed and processed for paraffin embedding. The glands were sectioned and stained for Foxp3 to identify regulatory T cells in the lymphocytic foci of (a) PBS treated and (b) BD-MSC treated mice. (c) The number of regulatory T cells per lymphocytic focus was then counted. Data are presented as means ± SEM (*n* = 3 for each group).

Foxp3 is specifically expressed in CD4^+^/CD25^+^ regulatory T cells (Tregs) which are critical cells in active suppression of autoimmune responses [13, 25]. The number of Foxp3^+^ Tregs was measured in lymphocytic foci in lacrimal gland sections from BD-MSC treated and PBS treated NOD mice. As shown in Figure 4 there was a modest, but not statistically significant, 21% increase in the number of Foxp3^+^ positive cells in the lymphocytic foci of mice treated with BD-MSCs when compared to those treated with PBS.

These data suggest that treatment with MSCs did not significantly increase the number of regulatory T cells in the lacrimal glands of NOD mice.

### 3.5. Aquaporin 5 Gene Expression Was Increased in BD-MSCs Treated NOD Mice

**Figure 5:**
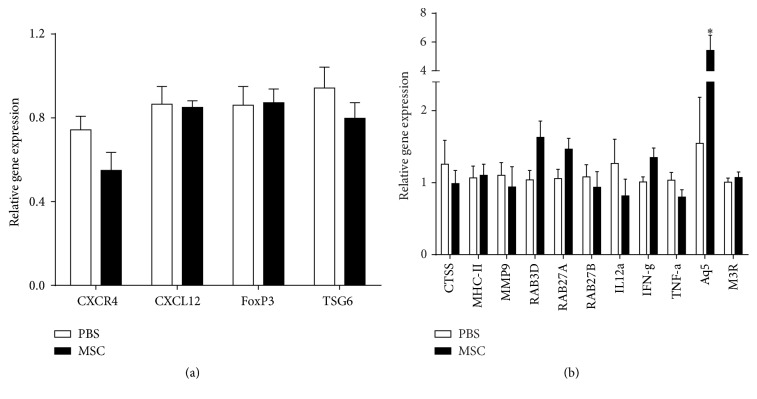
Gene expression of pro- and anti-inflammatory modulators in BD-MSC treated and nontreated NOD mice. Following 4 weeks of treatment total RNA was extracted from the lacrimal glands and used for (a) reverse transcriptase polymerase chain reaction of CXCR4, CXCL12, Foxp3, and TSG6 genes or (b) quantitative polymerase chain reaction of cathepsin S, MHC-II, MMP9, Rab3D, Rab27A, Rab27B, IL-12*α*, IFN-*γ*, TNF-*α*, aquaporin 5, and M3R genes. Data are presented as means ± SEM (RT-PCR, *n* = 10 for each group; qPCR, *n* = 7 for each group). *∗* denotes statistically significant difference compared to control.

Next, we conducted reverse transcriptase PCR experiments to investigate changes in gene expression of Foxp3, CXCL12, CXCR4, and TSG-6, since these genes are naturally involved in modulation of inflammation [12, 26–28]. In addition, expression of several genes known to be dysregulated in Sjögren's syndrome: CTSS, MHC-II, MMP9, Rab proteins (Rab3D, Rab27A, and Rab27B), IL12*α*, IFN-*γ*, TNF-*α*, and aquaporin 5 (Aq5), and the cholinergic muscarinic M3 receptor (M3R) were quantitatively measured using qPCR. As shown in Figure 5, we did not observe any differences in the expression of genes that are normally involved in modulation of inflammation, genes of proteins (Rab) involved in vesicular trafficking, or of proinflammatory genes. However, and as shown in Figure 5(b), there was a statistically significant increase in the gene expression of Aq5 (5.42 ± 1.06 versus 1.55 ± 0.64, *p* = 0.013) in the lacrimal glands from BD-MSC treated animals versus those from PBS treated animals. We performed immunostaining using anti-aquaporin 5 and Rab3D antibodies but could not detect a reliably significant increase in staining (data not shown).

These data suggest that increased gene expression of the water channel aquaporin 5 might be responsible for the increased tear production obtained following BD-MSC treatment.

## 4. Discussion

Sjögren's syndrome is a chronic autoimmune disorder of the exocrine glands characterized by inflammation and lymphocytic infiltrates of the affected glands [29]. MSCs from different sources have been successfully used to alleviate experimental and clinical Sjögren's syndrome symptoms [12, 13, 30, 31]. Notably, several studies reported therapeutic benefits of infused MSCs even though long-term engraftment of these cells could not be detected [32, 33]. MSCs derived from the bone marrow mainly act through the production of soluble trophic factors [32–34] and are involved in tissue repair due to their immunosuppressive and immunomodulatory functions [6, 9, 10].

In a recent study, it has been demonstrated that, following intravenous injection of human MSCs during corneal transplantation, immune rejection was decreased and allograft survival was prolonged [35]. Most of the intravenous infused MSCs were entrapped in the lungs and very few MSCs were found in the corneas after 28 days [35]. Indeed, several studies have reported that only 0.001–0.1% of systemically administered MSCs home to and engraft in sites of inflammation [35–37]. In our study we were unable to identify GFP labeled MSCs in the LG following 4 weeks of treatment. However, it is possible that MSCs did home to the LG in the short term but did not engraft into the tissue. Another study reported that topical MSC therapy in experimental dry eye syndrome improved tear volume and tear film stability [38]. In addition, labeled MSCs were observed in the meibomian glands and conjunctival epithelium along with increase in the number of secretory granules and number of goblet cells [38]. A different study using BD-MSCs in Sjögren's syndrome-like female NOD mice and human patients showed that the salivary gland secretory function was restored in both mouse models and Sjögren's syndrome patients [12]. In this study, we showed that mouse BD-MSCs significantly improved tears production, reduced the magnitude of lymphocytic infiltration in the NOD mouse model of Sjögren's syndrome, and increased the expression of the water channel aquaporin 5.

Tregs are key mediators of peripheral immunological tolerance and their absence results in excessive multisystem autoimmunity in mice and human [39, 40]. Foxp3 is a transcription factor that is specifically expressed on Tregs and is a master control gene for the development and function of CD4^+^/CD25^+^ Tregs [41]. Interestingly, it has been shown that, infusion of MSCs decreased proinflammatory T cell responses and increased anti-inflammatory T cell responses [12]. IL-10 and TGF-*β* are anti-inflammatory cytokines secreted by Tregs and they may play a role in mediating MSC responses and accelerating lacrimal gland repair [35, 42, 43]. However, in our study, the number of Foxp3^+^ Tregs that were found in the inflammatory lesions was not statistically significantly increased in BD-MSC treated lacrimal glands compared to PBS treated groups. These observations suggest that improving lacrimal gland function through BD-MSC treatment in our study does not involve modulation of the inflammatory responses. However, our pathological analyses showed that although the number of lymphocytic foci did not decrease, the size of these foci was significantly decreased following injection of BD-MSCs, which could be an indication of attenuated inflammation.

SDF-1/CXCR4 axis is essential in directing BD-MSCs to migrate toward inflammatory sites to control autoimmunity [12, 44]. SDF-1 (CXCL12) ligand and its receptor, CXCR4, are produced during chronic inflammation and play important roles in formation and maintenance of tertiary lymphoid tissues [45]. Interestingly, infused MSCs migrate toward the inflamed area in a SDF-1 dependent manner and blockade of CXCR4 in BD-MSCs with specific antibody could revoke the immunoregulatory activity and consequently the therapeutic effects of normal BD-MSCs [12]. In our study, we could not detect significant changes in the gene expression levels of SDF-1 or CXCR-4 following BD-MSC treatment. Similarly, we did not detect any significant changes in the expression of several proinflammatory genes (MHC-II, TNF-*α*, IL-12a, and MMP9).

Lacrimal gland protein secretion (exocytosis) is regulated in part by a family of Rab proteins which belong to the Ras superfamily of low-molecular-weight GTPases. Of these, Rab3D, Rab27a, and Rab27b have been shown to mediate regulated exocytosis in the lacrimal gland [46–48]. In addition, lacrimal gland fluid secretion is mediated, in part, by the water channel, aquaporin 5 [49]. The expression/localization of Rab proteins and aquaporin 5 has been shown to be altered in Sjögren's syndrome [50–52]. Our data showing increased expression of Rab3D, Rab27A (although not statistically significant, *p* = 0.053 and 0.062, resp.), and aquaporin 5 (*p* = 0.013) following injection of BD-MSCs might explain the effect of this treatment on improving lacrimal gland secretion.

In conclusion, our findings show that a single injection of BD-MSCs improved the secretory function of the lacrimal gland in Sjögren's syndrome-like diseased mice. This improved production of tears was most likely due to decreased inflammation (size of lymphocytic foci) and increased expression of the water channel aquaporin 5. Additional experiments are needed to further characterize the downstream targets involved in the improved lacrimal gland function in response to MSC delivery.

## Figures and Tables

**Table 1:** List of primers used in reverse transcriptase polymerase chain reaction.

Gene name	Primers	Amplicon size (bp)
Foxp3	F: GGC AAT AGT TCC TTC CCA GAG T	101
	R: ATG GCC CAT CGG ATA AGG GTG	
CXCL12	F: TGC CGC ACT TTC ACT CTC G	254
	R: TCA GCC GTG CAA CAA TCT GA	
CXCR4	F: TGT TGC CAT GGA ACC GAT CA	123
	R: TGG TGG GCA GGA AGA TCC TA	
TSG-6	F: AAT CCG GCT CAA CAG GAG TG	236
	R: CAT AGT CAG CCA AGC AGC CT	
GAPDH	F: ACC ACA GTC CAT GCC ATC AC	452
	R: TCC ACC ACC CTG TTG CTG TA	

**Table 2:** List of primers used in quantitative polymerase chain reaction.

Gene name	Cat # (ThermoFisher Scientific)	Amplicon size (bp)
CTSS	Mm01255859_m1	75
MHC-II	Mm00439216_m1	112
MMP9	Mm00442991_m1	76
Rab3D	Mm01151273_mH	94
Rab27A	Mm00469997_m1	61
Rab27B	Mm00472653_m1	66
IL-12a	Mm00434165_m1	68
IFN-gamma	Mm01168134_m1	100
TNF-alpha	Mm00443258_m1	81
Aq5	Mm00437578_m1	60
M3R	Mm00446300_s1	64
GAPDH	Mm99999915_g1	109
